# Hydrophobic Thin Films from Sol–Gel Processing: A Critical Review

**DOI:** 10.3390/ma14226799

**Published:** 2021-11-11

**Authors:** Matteo Poddighe, Plinio Innocenzi

**Affiliations:** Laboratory of Materials Science and Nanotechnology (LMNT), Department of Biomedical Sciences, University of Sassari, CR-INSTM, Viale San Pietro, 07100 Sassari, Italy; m.poddighe16@studenti.uniss.it

**Keywords:** sol–gel process, hydrophobic, wettability, contact angle, surfaces

## Abstract

Fabrication of hydrophobic thin films from a liquid phase is a hot topic with critical technological issues. Interest in the production of hydrophobic surfaces is growing steadily due to their wide applications in several industrial fields. Thin films from liquid phases can be deposited on different types of surfaces using a wide variety of techniques, while the design of the precursor solution offers the possibility of fine-tuning the properties of the hydrophobic coating layers. A general trend is the design of multifunctional films, which have different properties besides being hydrophobic. In the present review, we have described the synthesis through sol–gel processing of hydrophobic films enlightening the main achievements obtained in the field.

## 1. Introduction

The control of surface wettability is a crucial technological issue for several fields, such as microelectronics, separation membranes, car windshields, self-cleaning surfaces, traffic indicators and biotechnology [1,2,3,4,5,6]. The leading technologies used for the deposition of hydrophobic/hydrophilic thin films and modification of surfaces include chemical vapour deposition (CVD) [7], laser ablation [8] and plasma treatments [9]. Superhydrophobic surfaces have different applications in self-cleaning, anti-icing, and anti-sticking applications.

Besides physical deposition techniques also the wet chemistry route, in particular sol–gel processing [10], has received great interest for the fabrication of highly controlled hydrophobic-hydrophilic systems.

**Figure 1:**
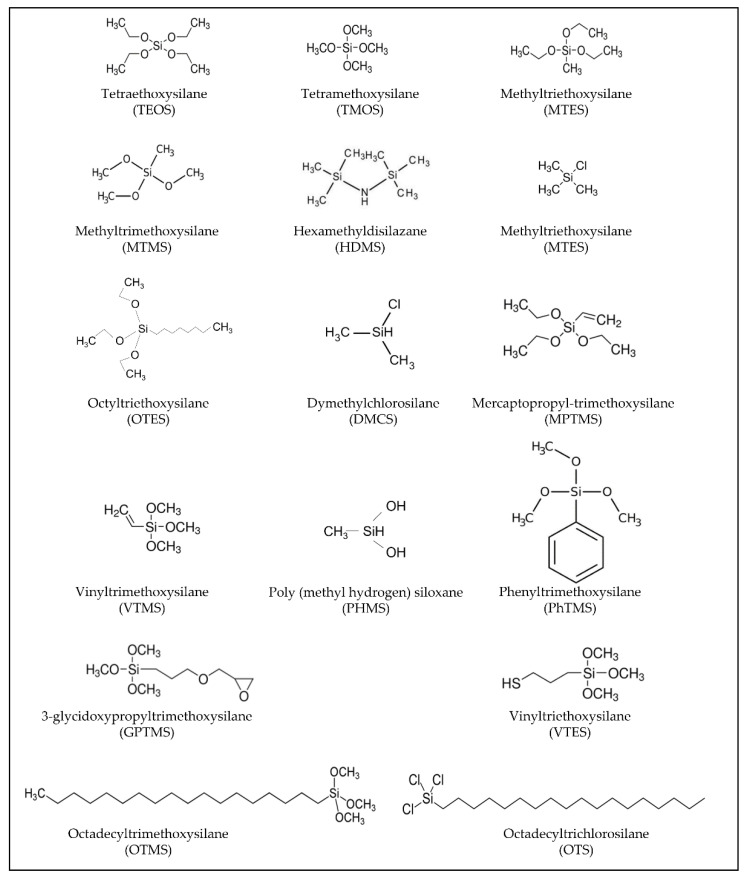
Nomenclature for all silanes reported in the review, including the corresponding abbreviations in brackets.

A class of compounds that are well suited for a fine design of thin films, including the wettability, are the alkoxides and organically modified alkoxides or organosilanes (Figure 1). They are employed as the reactive compounds for sol–gel chemistry to prepare functional thin films but are also used as surface modifiers and primers. The organically modified alkoxides are characterized by one or more covalent Si-C bonds that do not react during the sol–gel reactions of hydrolysis and condensation [11]. They can be applied for modifying the surface of different types of materials, e.g., glass or silicon wafers, from hydrophilic to hydrophobic and vice versa, through the chemical and physical tailoring of the surfaces [12]. An advantage of the solution processing route is the possibility of achieving a highly tailored coating design in terms of composition, thickness, surface roughness, contact angle, and mechanical properties. Thin layers can be simply applied on a wide variety of surfaces, i.e., ceramics, glass, metals, and polymers of different shape and compositions. The film can be deposited using several deposition techniques, such as dip-coating, spin-coating, doctor blade, and spray-coating, just to name the main one [13,14]. The composition of the functional coating can be largely adjusted by controlling the sol–gel chemistry. Alkoxides or organically modified alkoxides can be used alone or in combination for preparing the precursor solution. Organically modified alkoxides, in particular, offer the possibility of adding specific functionalities to the coatings by the organic modifier, such as epoxy [15], methyl, or aminic groups. Playing with composition, amount, and type of functional organic groups, thin films with specific hydrophilic-hydrophobic properties have been developed and applied in several fields [16,17]. The development of these functional coatings needs careful control of the mechanical properties and chemical durability [18,19].

On the other hand, deposition of thin films via solution processing has the advantage to allow a very fine design of the functional layer–substrate systems.

### 1.1. From Sol–Gel Chemistry to the Functional Coatings

Alkoxides are at the ground of sol–gel processing, widely studied and applied to deposit thin films on different surfaces. Sol–gel coatings can play a functional role, for instance, in photonics and microelectronic, but can also be simply applied as surface modifiers. Sol–gel methods have been employed for producing nanomaterials, micro and nanoparticle, [20,21,22,23,24], due to the simplicity of synthesis, the possibility of controlling particle size, the control of particle order and distribution, the possibility of obtaining both dense and porous structures under mild reaction conditions [11].

**Figure 2:**
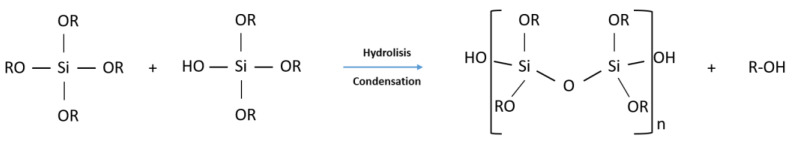
Illustration of the reaction of silicon alkoxides to form a silica network. R = alkoxy.

The phase transition from a sol to a gel, which is not a thermodynamic event, is governed by the hydrolysis and condensation reactions leading to the formation of an *interconnected network* (Figure 2) [25].

**Figure 3:**
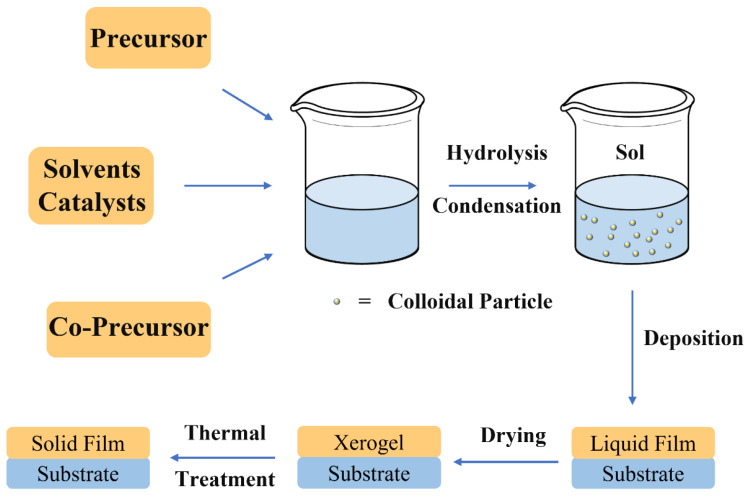
Diagram of the main steps in a sol–gel process.

The sol–gel process can be described in a simplified and general way as soft-chemistry route to prepare oxide and hybrid organic-inorganic materials from low-temperature processing. The synthesis can be described as a multistep method involving sequential synthesis steps (Figure 3):

➢ First step: Sol formation. A sol is obtained by mixing the precursors in water, organic solvent (e.g., methanol or ethanol) with a proper catalyst;
➢ Second step: Gel formation. The proceeds of the hydrolysis and condensation reactions allows the sol to gel transition with the formation of two continuous and interconnected phase, one liquid (the sol) and the other solid (the inorganic or hybrid network). This step is used only if a bulk must be obtained. In the case of a film deposition sol of controlled aging are employed. Aging governs the dimensions and structure of inorganic clusters in the precursor sols;
➢ Third step: Film deposition. The precursor sols are used to deposited thin films on different substrates via a specific deposition method (i.e., spin or dip-coating);
➢ Fourth step: Drying. After film deposition the residual solvent is removed through a drying process to obtain a xerogel;
➢ Fifth step: Firing. The film is condensed using a controlled heat-treatment to increase its density and mechanical properties. This step must be carefully controlled to avoid cracking or delamination from the substrate.

These advantages make it also possible to fabricate organic-inorganic hybrid surfaces where the organic compounds are bonded to an inorganic matrix. Optical waveguides, optical limiters, optical switches have been obtained using hybrid organic-inorganic coatings [26].

In a hybrid film, the organic component plays an important role because it changes the functional properties of the material and affects its mechanical properties and durability. It has been observed that as the length of the organic chain increases, the mechanical and adhesion properties of the film decrease [27].

### 1.2. Properties Design

The surface energy, the contact angle and the surface roughness are the key parameters to keep under control for the fabrication of hydrophobic thin films.

There are several ways to calculate the surface energy of a film, such as Berthelot method, Antonow method, Zisman approach, Good and Garifalco approach, Owens model, Neaumann model and Fowkes model [28,29,30]. Although there is still a heated debate about the validity of the methods, the most commonly used is the Fowkes method [31]. In the simplest case, for a solid-liquid system, the associated surface energy is given by:

(1) $$\gamma_{s}\;=\;\gamma_{s}\;+\;\gamma_{l}\;-\;2\;\sqrt{{\gamma_{s}}^{d}\;+\;{\gamma_{l}}^{d}}$$

where γ_s_ is the surface tension of the solid, γ_l_ is the surface tension of the liquid, γ_s_^d^ is the surface tension including the London forces associated with the solid and γ_l_^d^ is the surface tension including the London forces associated with the liquid.

**Figure 4:**
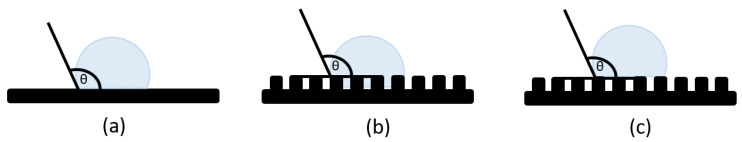
Scheme representing the three contact angles, (**a**) Young regime, (**b**) Wenzel regime, (**c**) Cassie–Baxter regime.

Measuring the contact angle is one of the fastest and most common methods for evaluating the hydrophobic-hydrophilic nature of a surface. The earliest studies of contact angle and surface wettability date back to the early 1800s, when Thomas Young attempted to explain his hypothesis about the factors influencing interactions at the interface between a smooth solid and a liquid [32]. Two other regimes were later formulated, the Wenzel [33] and the Cassie–Baxter [34] regimes (Figure 4).

A surface is considered hydrophobic when the contact angle between the water droplet and the surface is greater than 90° and superhydrophobic when the contact angle is greater than 150°.

A classic example of Young’s regime is that proposed by C. Lv et al. with a hydrophobic film fabricated by photolithography and etching of inductively coupled plasma [35]. T. Han et al. reported a work on the synthesis of a titanium oxide film modified with fluorinated reagents, with the aim of studying the structural modification of the surface to obtain a roughness belonging to the Wenzel regime [36]. A study validating the Cassie–Baxter regime has been reported by E. Bormashenko et al. [37], who studied the effect of three different micro-texturing of polymers on metal surfaces.

One of the main parameters affecting the hydrophobicity of a surface is roughness. The investigation of surface morphology has led to the discovery of the importance of roughness in the hydrophobic properties of an interface. A well-ordered structure with different degrees of porosity leads to an increase in the hydrophobic character due to the amount of air between the surface and the water droplet [38].

**Figure 5:**
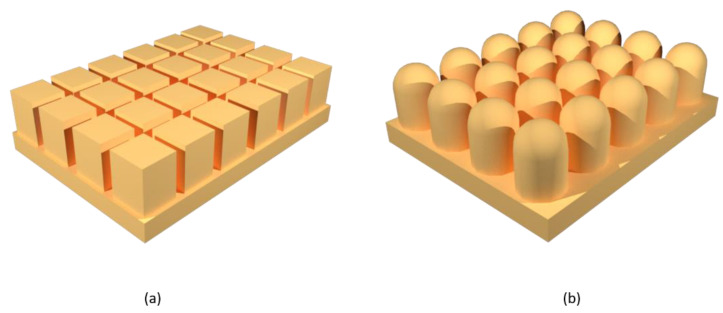
Representation of (**a**) flat-top and (**b**) semicircular structures.

In general, it is possible to convert the hydrophilic character of a surface into a hydrophobic one applying the proper structural modification [39]. A. Marmur et al. have described an ideal method for designing a hydrophobic surface [40]. In their work, they have studied the parameters that affect the hydrophobicity of the surface, taking into account mainly two morphological models (Figure 5): the flat-top and the semicircular. Both models follow the Cassie–Baxter regime. In the flat-top model, the water droplet does not enter between the pillars but stays on their surface, thanks to the air trapped at the interface. In the semicircular model, the pillars have a rounded shape, which causes the water droplet to flow over them, penetrating the structure and decreasing the hydrophobic character.

**Figure 6:**
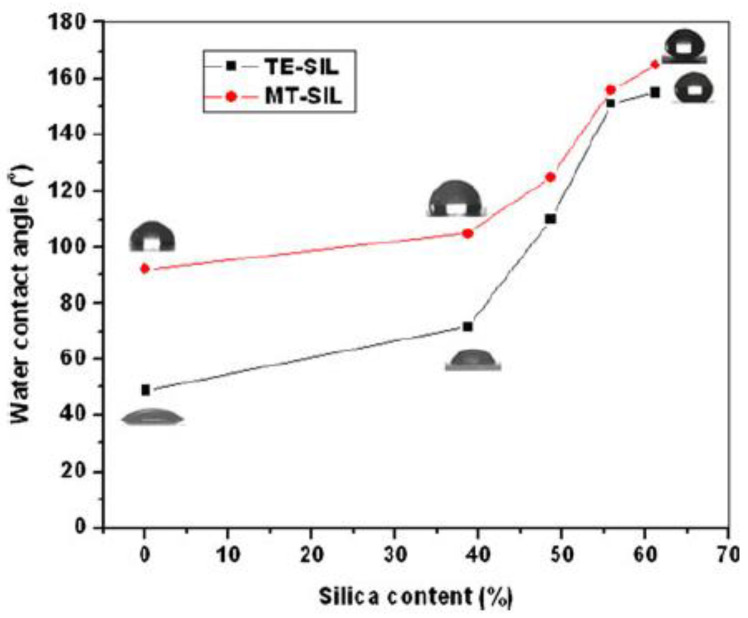
Effect of the concentration of hydrophobically modified silica nanoparticles on the water contact angle of sol–gel composite coatings prepared with sols of TEOS and MTES. Reproduced with permission from [41].

B. J. Basu and co-workers [41] obtained an interface with a crater-like roughness using hydrophobic silica nanoparticles (hexamethyldisilazane (HMDS)—treated fumed silica) in sols of TEOS and MTES. The films, deposited by spray or dip-coating, have an irregular rugosity as a function of silica nanoparticle content. Interestingly, the concentration of the silica nanoparticles affects the hydrophobicity (Figure 6). However, only the MTES composite films are crack-free and remain superhydrophobic (160° contact angle) up to thermal treatments of 400 °C, when the methyl groups degrade.

**Figure 7:**
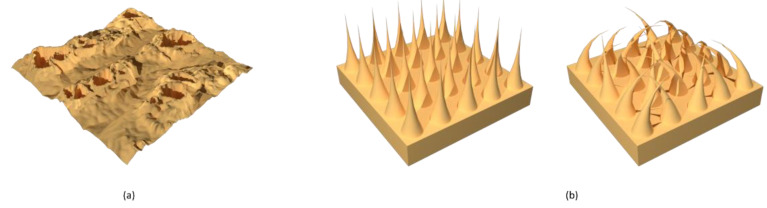
Representation of (**a**) crater-like morphology, (**b**) needle-like morphology.

The needle-like structure is the best morphology to achieve maximum hydrophobicity, but the poor mechanical strength does not make it worthwhile for real applications. (Figure 7) [42].

Several different approaches have been used to deposit thin films via sol–gel processing with the purpose of controlling its hydrophobic-hydrophilic properties. One of main differences is due to the deposition technique, they can be single or multiple steps via spin-coating, dip-coating or spray-coating. Moreover, the sol can incorporate nanoparticles whose surface can be also modified; the colloidal solution is then used for the deposition of hydrophobic films.

## 2. One-Step Surface Modification

**Figure 8:**
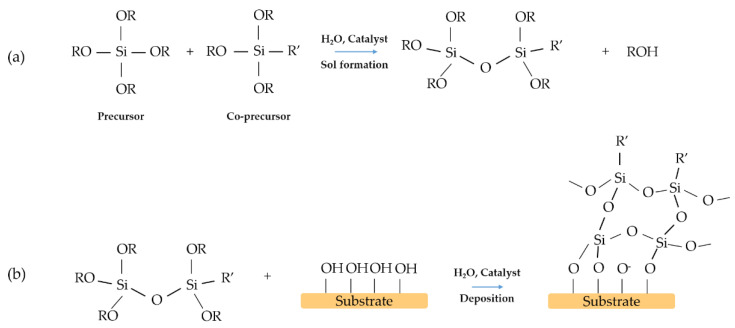
Example scheme of a one-step reaction: (**a**) preparation of the sol, (**b**) deposition of the film on the substrate. R and R’ organic groups. R’ is the functional group covalently bonded to silicon.

The one-step deposition of functional layers via solution processing is the simplest method and from the industrial point of view has several advantages as reduces the processing time and complexity of the process. It also offers the possibility of large-scale implementation. In general, a hybrid organic-inorganic single layer is enough to modify the contact angle of the material. The precursor is obtained by controlled co-hydrolysis and condensation of different alkoxides to obtain a homogeneous and transparent sol [43]. The resulting film is usually composed of a silica network modified with an organic group, R’, which could play the role of the hydrophobic moiety of the interface (Figure 8).

**Figure 9:**
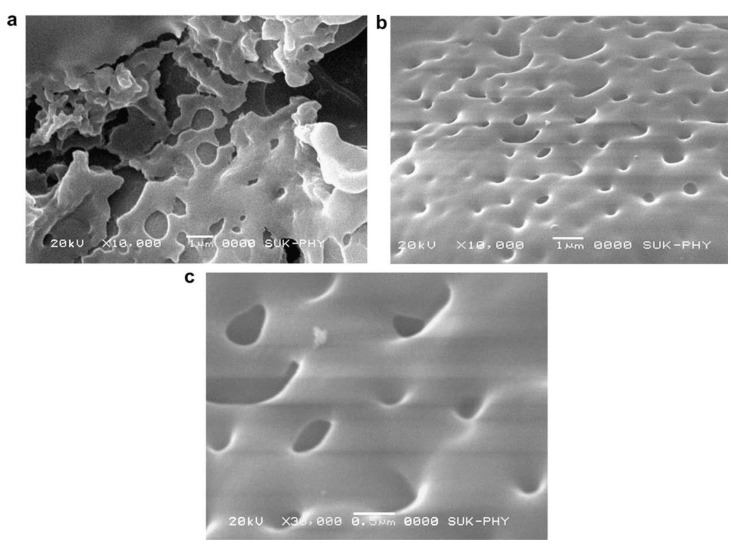
FESEM micrographs of (**a**) unmodified silica film and modified silica film at magnification of (**b**) 10,000× and (**c**) 30,000×. Reproduced with permission from [47].

A superhydrophobic hybrid film has been obtained by a one-pot synthesis, using MTES and PhTMS as precursors. Methyl and phenyl are the silica network modifiers. The precursor sol results from the mixing of MTES, methanol, water, and PhTMS which is added in a second step to react with MTES. The resulting sol has been deposited on a glass substrate via dip-coating. A thermal treatment at 150 °C is enough to densify the coating. It is important to note that the thermal treatment temperature is a critical parameter because it should be high enough to densify the films without degrading the organic groups. This MTES-PhTES layer reaches a very high contact angle, 164°, showing a superhydrophobic character [44]. Superhydrophobic coatings can be also obtained using hybrid TEOS-VTMS organic-inorganic precursor sols (contact angle 144°) [45] and TMOS-TMCS (contact angle 120°) [46]. Both the sols have been synthesised employing synthesis similar to those described above and have been deposited by dip-coating on a glass substrate. The effect of the functional groups (phenyl, vinyl, and methyl in PhTMS, VTMS and TMCS) leads to porous structures with a different degree of order (PhTMS pore size 200–1300 nm, VTMS pore size ~1000 nm, TMCS pore size 100–500 nm). The presence of pores (Figure 9) within these films increases the amount of air at the interface with the water droplet, thus limiting the contact with the surface. In these cases, the hydrophobic character of the surface increases.

Table 1 lists the main results reported in the literature for one-pot syntheses of hydrophobic surfaces. The technique has been employed on different substrates and using several deposition techniques, confirming the versatility of the technique.

## 3. Two-Steps Surface Modification

**Figure 10:**
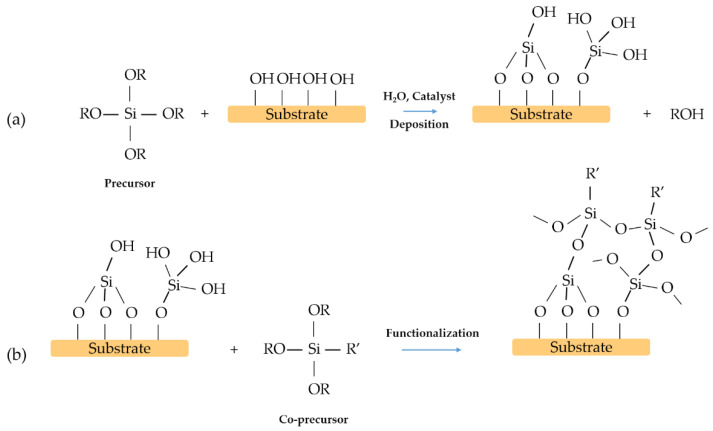
Example scheme of a two-step process: (**a**) deposition of the film, (**b**) functionalisation of the film.

This process consists of coating the substrate using two different layers. Generally, the first one is silica and the second one is a hybrid coating (Figure 10). Silica films obtained by sol–gel processing are, in general, hydrophilic. The number of residual hydroxyls, which results from the synthesis and thermal treatment of the silica films, control the contact angle [46].

The OH groups can be used to modify the silica surface, for instance, attaching specific functional groups.

Although more steps are required compared to a single layer deposition, the two-stage process allows obtaining a controlled functional layer with a highly homogeneous distribution of the organic functional groups over the entire surface, modifying the morphology of the interface and increasing the thickness and roughness [55].

With a two-step approach, films with high hydrophobic properties have been obtained, for example a MTMS-TMCS hybrid film. The precursor sol has been prepared by mixing MTMS, methanol, and water, and has been catalysed by NH_4_OH. A glass substrate is then immersed in the sol by dip-coating for 12 h at room temperature. The resulting film is heat-treated at 150 °C to densify the hybrid network. The sample is then functionalised by dipping the substrate in a 10% solution of TMCS in hexane followed by annealing at 100 °C, obtaining a superhydrophobic surface with a contact angle of 170° [56]. In this case, the high density of TMCS produces a high roughness, increasing the amount of air between the surface and the water droplet, thus improving the hydrophobic character of the film. In a similar way, a hydrophobic film can be obtained from a TEOS sol. The silica surface is then functionalised with OTS, in the presence of an acid catalyst and deposited on a glass substrate by dip-coating. The resulting film presents a contact angle of 120° [57].

An interesting study reports the influence of the functional group on the hydrophobic character of the surface [58].

**Figure 11:**
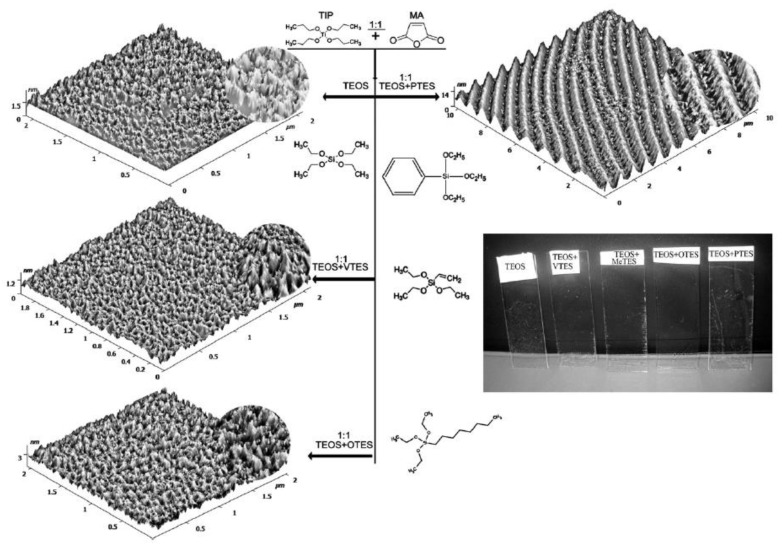
AFM images of the silica films prepared with different precursors and photo of the films deposited on glass slides, respectively. Reproduced with permission from [58].

A TEOS derived silica layer (74° contact angle) is functionalised using four different organically modified alkoxides, MTES, VTES, PhTES, and OTES, deposited on a glass substrate; titanium(IV) isopropoxide is used as a crosslinking agent. It was observed that longer is the length of the functional group (MTES < VTES < PhTES < OTES), more the hydrophobic character of the film increases, ranging from an 85° contact angle (TEOS-MTES system) to a maximum of 107° (TEOS-OTES system) (Figure 11). It was also noted that the contact angle of TEOS-VTES and TEOS-PhTES coatings were similar (92° and 91°, respectively) due to the analogous interaction of the C=C double bond and the aromatic ring with the hydroxyl groups at the interface, which is only partially condensed.

Table 2 lists some of the main two-step syntheses for producing hydrophobic silica films.

## 4. Multilayer Films

**Figure 12:**
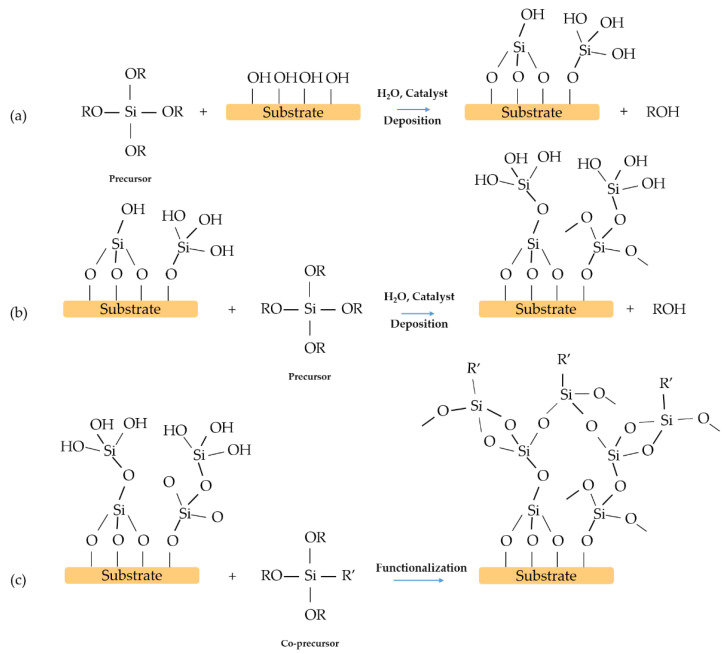
Example scheme of a multilayer synthesis: (**a**) deposition of the first layer, (**b**) deposition of the second layer, (**c**) functionalisation of the film.

This method of synthesis consists of making several layers to modify the morphology of the surface increasing the roughness of the film and consequently the hydrophobicity of the interface (Figure 12). This method has not been extensively applied to the synthesis of hydrophobic films, likely because increases the complexity of the process and reduces the technological appeal. However, it represents an important route if multifunctional coatings must be fabricated.

The use of polymerising compounds is a technique often used in the synthesis of hydrophobic films. An interesting work reports that the addition of polyacrylic acid (PAA) in a TEOS sol, allows obtaining layers with high roughness. In this case, an acid catalysed silica sol (TEOS in ethanol), is pre-reacted before the addition of PAA. Then, a second basic sol is prepared with TEOS, NH_4_OH, and ethanol, without the addition of the polymerising agent. The two sols, after aging for 24 h, are mixed and deposited on a glass substrate via dip-coating. The film is then air-dried for a few minutes. This procedure is repeated five times, with a final thermal treatment at 500 °C for 30 min. The interface is modified by chemical vapour deposition. The substrate is placed inside a sealed vessel containing TMCS, with no direct contact between solid and liquid. The vessel is then put in an oven at 140 °C to allow the reaction between the OH groups of the substrate and the TMCS vapour. Finally, the substrate is sonicated and dried under a flow of N_2_. The resulting film shows a contact angle of 151° [64].

The strategy of producing different sols is particularly feasible to fabricate multilayer systems. For example, alternating the deposition on a glass substrate by dip-coating, of a TEOS sol and a TEOS-PEG200 sol, with final functionalisation with TMCS, makes it possible to obtain films that present dense (inner layer)-porous (outer layer) hybrid structures, with high roughness [65]. A film of this type shows a contact angle of 135°. A triple-layer film is obtained using the same approach, using TEOS and tetrabutyl ortho-titanate (TBOT). The use of TBOT makes it possible to increase the mechanical properties of the surface, increasing its resistance to abrasion and adhesive strength to the substrate. In both tests, no change in film transmittance has been observed, indicating the high mechanical properties of the developed surface. In this case, a glass substrate is dip-coated in three different sols, one of TEOS, one of TBOT and one of TEOS-TBOT, and finally functionalised with HMDS to obtain hydrophobic properties. This triple layer has resulted in an hybdrophobic abrasion-resistant and antireflective coating, making it ideal in the field of solar cells [66].

## 5. Hydrophobic Film with Modified Nanoparticles

**Figure 13:**
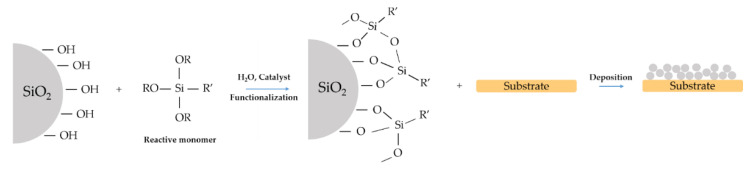
Example scheme of a synthesis of a film with modified nanoparticles.

Another way of achieving a hydrophobic film is using modified nanoparticles (Figure 13). Nanoparticles can be arranged in a well-ordered structure, resulting in superhydrophobic surfaces [67].

**Figure 14:**
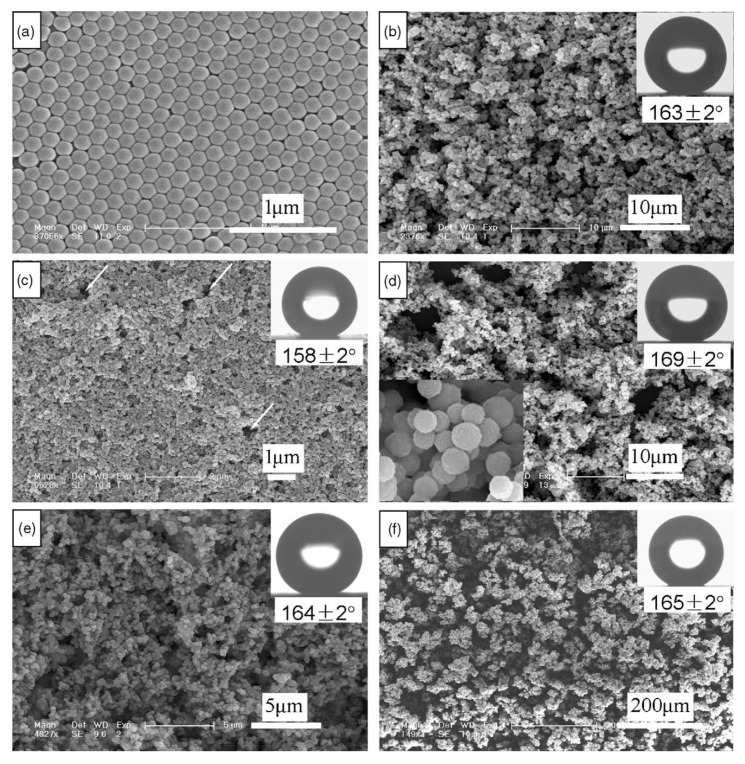
FESEM micrographs of (**a**) the unmodified silica nanoparticles with diameters of 167 nm and the vinyl-modified silica nanoparticles with different diameters: (**b**) 85 nm, (**c**) 167 nm, (**d**) 360 nm, (**e**) 453 nm, and (**f**) 1600 nm. The insets on the top right corners are the corresponding profiles of water drops on the surfaces and the water contact angles. The inset on the bottom left of (**e**) shows the enlarged particles. Reproduced with permission from [68].

The modification of silica nanoparticles with vinyl functional groups allows obtaining films with a high contact angle of 169°. To prepare the hydrophobic layer, 1 g of Stöber silica nanoparticles (diameter of 453 nm) is dispersed in ethanol with an excess of VTEOS, to substitute all the hydroxyls with vinyl groups. Then ammonia is added, and the final mixture is left to react under stirring. The modified nanoparticles are then washed and dispersed again in ethanol to obtain the final sol. The vinyl-modified silica nanoparticles are used to obtain a film on a wafer substrate by drop casting. The size of the nanoparticles, which ranged from 85 to 1600 nm, does not affect the contact angle when they are 150–1600 nm in the range, while for particles smaller than 150 nm, the hydrophobic character decreases [68] (Figure 14).

The addition of a polymer, such as polymethylmethacrylate (PMMA), allows the obtaining of a nanocomposite film, with a porous structure and high roughness, by incorporating silica nanoparticles within the PMMA polymer matrix. This leads to an increase in the abrasion resistance of the film and the achievement of a contact angle of 165° [69]. By combining two sols, for example one of acid-catalysed TEOS and one of base-catalysed TEOS/MTES, deposited on a silicon wafer substrate by dip-coating, it is possible to obtain hydrophobic bilayer films (contact angle 115°) with methyl-modified silica nanoparticles [70].

This approach makes it possible to increase the optical properties of the surface. By modifying the silica nanoparticles with OTS and depositing the film on a silicon wafer substrate by dipping, hydrophobic surfaces with a contact angle of 119° and high tribological properties have been fabricated [71]. Other results in the production of hydrophobic films using modified silica nanoparticles are shown in Table 3.

## 6. FAS (Fluoroalkylsilanes)

A possible strategy to obtain superhydrophobic surfaces is fabricating rough surfaces modified with low surface energy molecules such as fluoroalkysilanes (FAS). The use of FAS has always been investigated because of the ease of obtaining hydrophobic, chemically and mechanically resistant surfaces, compared to classical alkylsilanes [87]. In recent years, however, the presence of fluorinated agents has banned their use because of their dangerous effect on human health [88]. In particular, acute lung inflammation has been observed in the operators during deposition of hydrophobic coatings via spray-drying [89].

Corrosion resistance is a property often required in coatings and with the use of FAS it is possible to achieve this type of film. An example is an aluminium alloy substrate that is dip-coated in a PFANI (polyfluoroaniline) modified GPTMS sol, resulting in a hydrophobic film with a contact angle of 113° [90]. Using PFAS, it is possible to obtain other films with a hydrophobic character (contact angle of 112°), for example by inserting them into silica and titanium oxide sols and then depositing them on fused silica by spin-coating [91].

## 7. A Brief Comparison of the Different Synthesis Routes

**Figure 15:**
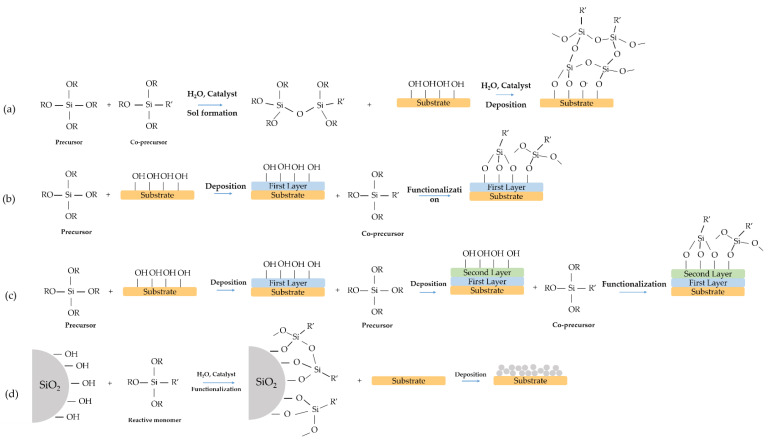
Schematic summary of all synthesis approaches reported in this review: (**a**) one-step synthesis, (**b**) two-step synthesis, (**c**) multilayer synthesis, (**d**) film with modified nanoparticles synthesis.

Fabrication of superhydrophobic coatings via sol–gel processing offers a wide spectrum of different solutions which can be customized as a function of the specific requirements. In many cases, the choice is dictated by the need for combining several functions in the thin films, in other is just the need for controlling the contact angle through a robust, simple, and low-cost process. Each of these synthesis routes (Figure 15) has its advantages and disadvantages, peculiar reaction conditions, chemical precursors and processing; in general, it is challenging to select the best method.

Looking at the techniques employing modified nanoparticles to obtain hydrophobic films, it can be said that they exhibit the most performing mechanical properties, with high abrasive resistance, strong adhesion, scratch, and corrosion resistance. The synthesis is, however, often very complicated, with several steps and treatments, such as separating the particles to obtain the desired size or preparing different sols to fabricate at first the nanoparticles, modifying them, and finally depositing on a substrate. The same situation occurs in the syntheses that employ several depositions steps. Multilayers are deposited on the same substrate to design the hydrophobic characteristics together with other functional properties. Even if from the technological point of view a multistep process is less appealing for producing hydrophobic coatings, it represents an important toll when multifunctional coatings are required.

On the other hand, one-pot and two-steps syntheses are simpler and faster, but often the hydrophobic films exhibit lower mechanical properties. The main advantages of one-pot and two-steps syntheses are the mild reaction conditions (sometimes the reaction takes place at room temperature), shorter aging times, reduced use of reagents and low-temperature heat treatments (no more than 200 °C). One way to increase the hardness and hydrophobicity of the films is to use a polymerising agent, such as PMMA, to modify the surface at the interface, giving strength to the structure. Considering a compromise between film quality and simplicity of synthesis, the latter two approaches are the most suitable for large-scale applications.

## 8. Conclusions

In this review, we have discussed the various approaches that can be followed to synthesise hydrophobic films using sol–gel processing. The sol–gel method is a very versatile technique, which allows obtaining mechanically and chemically resistant hydrophobic films. Although surface modification with fluoroalkysilanes has been one of the most successful ways to control the surface energy, currently their use is severely restricted due to the potential toxicity to human health. This limitation has prompted increased research into new modifying reagents and methods, looking for feasible solutions. Organosilanes with hydrophobic functional groups represent an attractive alternative.

In each synthesis route the general strategy is to modify the morphology of the film surface increasing the roughness, while the surface energy of the interface, is modified by replacing polar hydrophilic species, such as hydroxyls, with apolar hydrophobic groups. Ideally, a compromise between these two factors should be reached by choosing the most suitable modifying agent and processing conditions.

Producing hydrophobic films using modified nanoparticles is undoubtedly of great interest; the modification of surface, dimension, composition, and loading of the nanoparticles in the precursor sol, represent a feasible toolbox for designing the film property. The overall synthesis, which requires several steps, does not currently make this method particularly attractive feasible for a large-scale production.

The most promising approaches appear to be one-step and two-step synthesis routes, which offers a good compromise in terms of performance and production costs. A clear trend is the fabrication of multifunctional coatings that require the combination of different properties in the same material. Superhydrophobicity must be combined with optical, mechanical, surface requirements which could be fulfilled only through a complex design of the material.

The various technological limitations still need further improvements to develop advanced methods for the production of hydrophobic surfaces. Depositions of films from a liquid phase, such as sol–gel, are largely employed in the industry but are always challenged by physical deposition methods. The flexibility of sol–gel synthesis offers, however, the possibility of a very fine tuning of composition and properties, which makes the method still highly attractive also for the deposition of hydrophobic-superhydrophobic coatings.

The greatest challenge in the next future is, however, moving to deposition methods which are “green”, using non-toxic reagents, water as solvent and fabrication methods which reduces the exposure of the operators to aerosols. The environmental issues must be considered to be a high priority to develop the materials for the future.

## Figures and Tables

**Table 1:** One-pot syntheses of hydrophobic silica films.

Silicon Alkoxide	Organically Modified Alkoxide	Substrate	Coating Method	Contact Angle/°	Data from Ref.
TEOS	methyl (MTES)	Glass	Dip-coating	160	[47]
TEOS	HDMS	Glass	Dip- and Spray-coating	166	[48]
TEOS	VTMS	Glass	Dip-coating	145	[49]
TEOS	TMES	Glass	Dip-coating	151	[50]
TEOS	phenyl	Glass	Dip-coating	133	[51]
TEOS	PMHS	PMMA	Dip-coating	125	[52]
TEOS	OTES	Glass	Dip-coating	125	[53]
TMOS	HMDS	Stainless steel plate	Dip-coating	145	[54]

**Table 2:** Two-step syntheses of hydrophobic silica films.

Silicon Alkoxide	Organically Modified Alkoxide	Substrate	Coating Method	Contact Angle /°	Data from Ref.
MTES/TMMS	TMCS	Glass	Dip-coating	172	[59]
MTES	TMCS	Glass	Spray-coating	167	[60]
TEOS	DMCS/TMCS	Glass	Dip-coating	162	[61]
TEOS	TMCS	Glass	Dip-coating	153	[62]
ETES	iso-OTMS	Glass	Dip-coating	160	[63]

**Table 3:** Hydrophobic modified silica nanoparticles.

Silicon Alkoxide	Organically Modified Alkoxide	Substrate	Coating Method	Contact Angle/°	Data from Ref.
TEOS	HDMS/MTMS	Glass	Spin-coating	165	[72]
TEOS	HMDS	Glass	Dip-coating	160	[73]
TEOS	HMDS	Glass and Silicon wafer	Dip-coating	126	[74]
TEOS	OTS	Glass	Spin-coating	150	[75]
TEOS	MTES/VTES/OTES/OTS	Glass	Brushing	146	[76]
TEOS	MTES	Stainless steel mesh	Dip-coating	142	[77]
TEOS	GPTMS/HDTMS	Silicon, glass, and cellulosic cotton	Spin-coating	141	[78]
TEOS	PMHS	Glass	Dip-coating	130	[79]
TEOS	MTES	Glass	Dip-coating	122	[80]
TEOS	TEOS	Glass	Spin-coating	121	[81]
MTES/TEOS	PDMS	Glass	Spray-coating	133	[82]
MTES/TEOS	PDMS	Glass	Spray-coating	120	[83]
HMDS	MTMS	Glass	Dip-coating	161	[84]
SiO_2_-NPs	MPTMS	Silicon wafer	Spray-coating	162	[85]
SiO_2_-NPs	PDMS/OTMS	Shrink film	Spray-coating	155	[86]

## Data Availability

Not applicable.
